# The Route of Early T Cell Development: Crosstalk between Epigenetic and Transcription Factors

**DOI:** 10.3390/cells10051074

**Published:** 2021-04-30

**Authors:** Veronica Della Chiara, Lucia Daxinger, Frank J. T. Staal

**Affiliations:** 1 Department of Human Genetics, Leiden University Medical Centre (LUMC), 2300 RC Leiden, The Netherlands; v.della_chiara@lumc.nl (V.D.C.); L.Clemens-Daxinger@lumc.nl (L.D.); 2 Department of Immunology, Leiden University Medical Center, 2333 ZA Leiden, The Netherlands

**Keywords:** T cell development, T cell commitment, transcription factors, epigenetic regulators, chromatin modifiers, Tcf1, Bcl11b, Dnmt3, Polycomb genes

## Abstract

Hematopoietic multipotent progenitors seed the thymus and then follow consecutive developmental stages until the formation of mature T cells. During this process, phenotypic changes of T cells entail stage-specific transcriptional programs that underlie the dynamic progression towards mature lymphocytes. Lineage-specific transcription factors are key drivers of T cell specification and act in conjunction with epigenetic regulators that have also been elucidated as crucial players in the establishment of regulatory networks necessary for proper T cell development. In this review, we summarize the activity of transcription factors and epigenetic regulators that together orchestrate the intricacies of early T cell development with a focus on regulation of T cell lineage commitment.

## 1. Introduction

Hematopoiesis, the formation of blood cells, is a well-studied developmental process, during which transcription factors and epigenetic regulators operate together in order to ensure faithful progression toward the production of mature blood cells [1,2]. Lineage restriction events reflect changes in the transcriptional programs and epigenetic regulation [3,4], and defects in these regulatory mechanisms can promote leukemogenesis [5,6]. These intrinsic programs require cell-extrinsic factors, such as signaling pathways, cytokines and growth factors, provided by a specific microenvironment. While most blood cell types develop in the bone marrow [7], specialized white blood cells differentiate in the thymus from multipotent progenitors that pass through different stages until they develop into mature T cells [8,9]. The complex regulatory mechanisms that enable this stepwise differentiation are governed by transcriptional and epigenetic programs [10,11]. Master transcription factors and epigenetic regulators drive the journey from early thymic progenitors to mature T cells by repressing alternative non-T cell fates and enhancing the dynamic progress towards lineage commitment. The roles of transcription and epigenetic factors can be elucidated in a stage-specific manner by using genetically modified mouse models and flow cytometry-based strategies. The interplay between transcriptional programs and epigenetic regulation has been well-characterized for the late differentiation steps (CD4 single positive versus CD8 single positive) and has been reviewed elsewhere [12,13,14,15]. However, less is known about the transcription factors and chromatin modifiers controlling early T cell development. In this review, we discuss the journey of early thymic progenitors toward committed T cells and the lessons learned from murine models about the crucial roles of key transcription factors and epigenetic regulators in the establishment of T cell identity.

## 2. Introduction of Epigenetics

**Figure 1:**
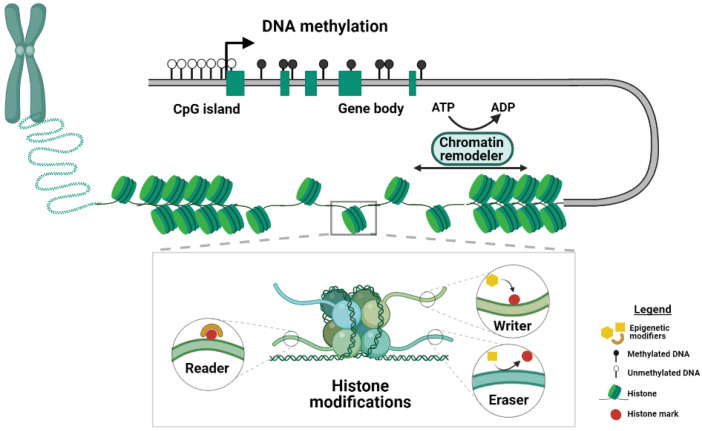
Schematic of the mechanisms of epigenetic regulation. DNA methylation, histone modifications and chromatin remodeling represent three different kinds of epigenetic mechanisms. Main players in the histone modification machinery are depicted in the inset. Proteins that covalently attach chemical groups to the histone tails are termed writers, whereas the so-called readers can recognize and bind to histone modifications. Enzymes that remove histone marks are termed erasers. DNA methylation is found across different genomic elements. For example, CpG islands, which are often found in the proximity of promoters, are usually depleted of methylation, whereas gene bodies are heavily methylated. The chromatin structure is controlled by chromatin remodeler complexes that use the hydrolysis of ATP to mediate the packaging of the chromatin. Created with BioRender.com.

Epigenetic regulation is key to the normal development of an organism because it allows for modulating gene expression levels through the addition of chemical modifications to the DNA and its associated histone proteins, which are referred to as epigenetic marks (Figure 1).

DNA methylation, which involves the addition of a methyl group to the 5th position of a cytosine in a CpG dinucleotide context, is one of the best-studied epigenetic marks and essential for mammalian development [16]. DNA methylation is mainly associated with gene silencing, and throughout the genome, 70–80% of CpG dinucleotides are methylated [17]. CpG-rich sequences near promoter regions, referred to as CpG islands, usually lack DNA methylation [18]. More recently, DNA methylation has also been associated with active transcription when found across gene bodies [19]. The de novo DNA methyltransferases (DNMTs), DNMT3A and DNMT3B, are responsible for the establishment of DNA methylation early in embryonic development [20], whereas DNMT1 is required for the maintenance of DNA methylation throughout cell divisions, thereby conferring heritable epigenetic memory [21]. In addition to passive loss of DNA methylation during cell divisions, DNA methylation can be actively removed by members of the ten-eleven translocation (TET) enzyme family. These so called “erasers” mediate DNA demethylation by oxidizing 5-methylcytosine to 5-hydroxy-methylcytosine [22].

Histone marks are post-translational modifications found at the amino-terminal tails of histone proteins and include acetylation, lysine and arginine methylation, phosphorylation and ubiquitination [23]. Different genomic features acquire different types of modifications, and histone marks can be associated with transcriptional activation and repression [24]. For example, both mono-/tri-methylation of histone 3 lysine 4 (H3K4me1/3) and H3K27ac are associated with active transcription but get deposited at different genomic elements. Whereas H3K4me1 and H3K27ac mark enhancers, H3K4me3 is mainly found at the promoters of actively transcribed genes [25,26,27]. In contrast, H3K9me3 and H3K27me3 are referred to as repressive marks, and while H3K9me3 is found in repetitive regions of the genome, transcriptionally silenced genes are enriched in H3K27me3 [28]. Additionally, the methylation of arginine residues can be either associated to transcriptional activation or repression [29]. Histone variants H2A and H2B are post-translationally modified, and the ubiquitination is one of the most frequent and studied tags. For example, monoubiquitination of histone H2A lysine 119 (H2AK119ub) is a repressive mark [30], whereas H2BK120ub is associated with actively transcribed genes [31]. The enzymes responsible for the deposition and removal of these marks are referred to as writers and erasers, respectively. In addition, the readers recognize specific modifications and can mediate downstream effects.

Organization and packaging of chromatin can be restructured by so-called chromatin remodeling complexes that use the hydrolysis of ATP molecules to modulate the degree of chromatin compaction [32] (Figure 1). This extra layer of epigenetic regulation allows rapid adjustment of chromatin states and plays a critical role during development [33]. Chromatin remodeling involves nucleosome repositioning, which influences chromatin accessibility and the binding of transcription factors to DNA [34]. Four subfamilies of chromatin remodelers have been identified: imitation switch (ISWI), chromodomain helicase DNA-binding (CHD), switch/sucrose non-fermentable (SWI/SNF) and INO80, according to the similarity between the ATPase domains [35,36]. ISWI and CHD remodeler subfamilies co-operate to assemble the nucleosome on the newly synthetized DNA. SWI/SNF subfamily enzymes use their DNA translocase activity to slide the octamers along the DNA, eject the nucleosome or remove histone dimers. The remodeling mediated by the INO80 subfamily factors is responsible for the exchange of canonical histone proteins and related variants. 

## 3. Overview of Mouse T Cell Development

T cells derive from lymphoid progenitor cells that develop in the fetal liver or adult bone marrow, migrate via the blood and seed the thymus at the cortical medullary junction [37,38]. Specialized thymic epithelial cells provide the necessary stimuli to activate the Notch signaling pathway that drives progenitor cells to develop into mature differentiated cells in a stepwise manner [39,40,41]. Mouse T cell development is marked by changes in the expression of surface molecules, subdividing T cells into double negative (DN), double positive (DP) and single positive (SP) populations, according to the expression of CD4 and CD8 coreceptors [42,43,44]. Mouse and human T cell development are homologous, but different markers distinguish DN stages in humans (compared in Reference [44]). However, here we focus on mouse T cell development because most of the knowledge on epigenetic and transcription factors comes from using genetically modified mouse models. Early T-cell progenitors (ETPs) are the most immature DN subpopulation, characterized by the expression of the receptor tyrosine kinase c-Kit (or CD117) and the hyaluronic acid receptor (CD44), but they lack surface expression of the IL-2Rα chain (CD25) [45,46,47]. This heterogeneous population is multipotent with the potential to generate other lineages, such as myeloid cells, B cells and natural killer (NK) cells [46,47,48]. As ETPs move towards the outer thymic cortex, CD25 expression increases, giving rise to CD44+CD25+ T cells, referred to as DN2. Based on the expression of CD117, DN2 cells can be further subdivided into DN2a and DN2b cells. CD117 is more highly expressed on the surface of DN2a compared to DN2b cells. These two substages define the transition from uncommitted to committed T cells, during which the Notch signaling-induced cascade mediates the repression of alternative lineage development [49,50]. Full T cell lineage commitment occurs upon transition to the CD44−CD25+ DN3 stage. CD27 expression discriminates between two subpopulations, DN3a (CD44−CD25+CD27−) and DN3b cells (CD44−CD25+CD27+), defined as pre- and post-β-selection cells respectively [51]. β-selection is a key developmental checkpoint at the DN3 stage wherein T cell receptor (TCR) rearrangement, mediated by the recombinant activating genes 1 and 2 (RAG1 and RAG2), begins [52,53]. After successful rearrangement of the TCRβ locus, the expressed β chains couple with the invariant pre-Tα receptor, forming the pre-TCR complex [54,55]. Cells with successful β-selection downregulate the expression of CD25 and become DN4 cells, which then progress to the DP stage through the immature single positive (ISP) cells expressing in mouse CD8, but not CD4 nor CD3 [56]. The development of DN3 cells with unsuccessful β-selection stops, and cells undergo apoptosis. At the DP stage, TCRα gene rearrangements initiate, and an αβ-TCR is produced [57]. Subsequently, T cells undergo positive and negative selection in the cortex. Positive selection is intended to select for T cells with a functional TCR, which means they are able to interact with the major histocompatibility complex (MHC) on cortical epithelial cells that function as antigen-presenting cells [58]. During the negative selection, the cells are screened for potential autoreactivity and will undergo apoptosis when a self-peptide is encountered in the MHC [59]. Thymocytes that respond to self-antigens are eliminated. Finally, the cells with a functional TCR differentiate into CD4+ helper or CD8+ cytotoxic T cells. These now-called single positive (SP) cell types can leave the thymus and emigrate to the periphery [60].

## 4. Key Transcription Factors in Early T Cells

**Figure 2:**
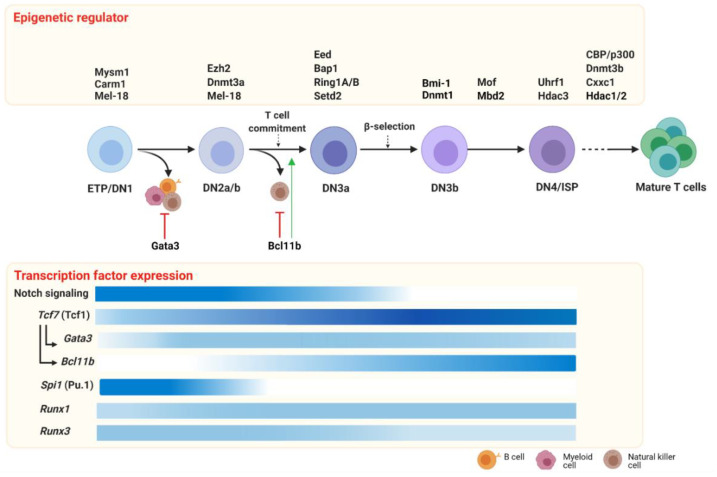
Epigenetic regulators and transcription factors driving early T cell development. In the thymus, T cell development, triggered by the Notch signaling pathway, requires the expression of key transcription factors (lower panel) and epigenetic modifiers (upper panel). During the first developmental stages, ETP and DN2 cells retain lineage plasticity, most likely sustained by non-T cell transcription factors such as Pu.1 and inhibited by T-cell factors such as Gata3 and Bcl11b (red arrows). The potential to develop myeloid, B and NK cells is shut down at the T cell commitment checkpoint during the transition to the DN2-DN3 stage, which is promoted by Bcl11b (green arrow). DN3b are selected after a successful TCR gene rearrangement (β selection) that, after the last immature stages (DN4/ISP), can progress toward mature CD4+ or CD8+ TCRαβ+ T cells. In the lower panel, the expression patterns of the key transcription factors of early T cells are indicated (the color’s intensity correlates with the gene expression level; darker color indicates a higher expression). Early T cell development is dynamically orchestrated by epigenetic regulators that contribute in shaping T cell survival and specification. In the upper panel, we report the epigenetic regulators at a specific stage or transition, during which the knockout murine models displayed a phenotype. Of note, conditional knockout models have often been employed to study epigenetic regulator function in T cell development. In those cases, it cannot be excluded that the requirement at other stages might exist, but this has not been investigated yet. Abbreviations: ETP: early thymic progenitors; DN: double negative; ISP: immature single positive. Created with BioRender.com.

T cell development is dynamically orchestrated by a core set of transcription factors establishing T cell lineage commitment (extensively reviewed in Reference [61]). The critical master regulators for T cell commitment are T cell factor 1 (Tcf1) [62,63], Bcl11b [64], Gata3 [65] and to a lesser extent Pu.1 [66] and the Runx family of transcription factors [67]. The T cell regulatory network is continuously changing throughout all the subsequent developmental stages within the thymus, starting from the most immature cells to the SP cell subsets. The Notch signaling pathway initiates the cascade of transcriptional program changes in multipotent and heterogeneous progenitor cells, which lose their competence for alternative fates and activate the specific T cell regulatory gene network throughout their development [48]. Here, we focus on the key transcription factors Tcf1, Bcl11b, Gata3, Pu.1 and Runx1/3 that have stage-specific patterns of expression (Figure 2) and together create a transcriptional network that regulates early T cell development.

Tcf1 (encoded by the *Tcf7* gene) is a master regulator of T-lineage specification and is activated directly by Notch signaling at the ETP stage [62,63]. The upregulation of *Tcf7* upon entry of lymphoid progenitors to the thymus is crucial for the progression toward subsequent developmental stages, and Tcf1 is required for the expression of essential T cells genes such as *Bcl11b* and *Gata3* [62,63]. The functional relationship between these major first transcription factors (Tcf1, Bcl11b and Gata3) that shape the T cell gene program has long been unclear. We recently unraveled the functional hierarchy of this core set of transcription factors in T cell commitment [68]. Our work and others showed that disruption of *Tcf7* resulted in abnormal gene expression profiles and chromatin accessibility in DN3 [68] and DP thymocytes [69]. Tcf1 bound to the *Gata3* and *Bcl11b* promoter/enhancer regions, where a more compact chromatin was observed in the absence of Tcf1 [68]. Motif analysis revealed that sites that displayed a differential chromatin opening were enriched for Tcf1 binding sites, suggesting that *Gata3* and *Bcl11b* were two direct Tcf1 targets in thymocytes [68], in accordance with previous studies from Rothenberg and colleagues [64,70]. The ectopic expression of *Gata3* and *Bcl11b* in *Tcf7*-deficient murine cells suggested a collaboration among the transcription factors: Gata3 suppresses B and myeloid fate in early T cells, whereas Bcl11b rescues impaired T cell development [68]. Interestingly, lack of Tcf1 is also associated with a high chance of developing leukemias [71], as Tcf1 is not only an active transcription factor when associated with the Wnt mediator β-catenin, but also a transcriptional repressor [72]. Collectively, these studies emphasize the role of Tcf1 as essential regulator of T cell development.

Gata3, a member of the Gata transcription factor family, is considered another key member of the T cell transcriptional network. Gata3 plays multiple roles in thymocyte development in a stage-specific manner, affecting T-cell survival, growth, commitment and progression into mature stages [65,73]. Notch signaling and Tcf1 induce the expression of *Gata3* that gradually increases from thymic progenitors to mature T cells [74,75,76]. Gata3, in response to Notch signaling, is required for the repression of diversion to non-T cell lineages, such as B cells and mast cells [68,76,77,78]. Gata3 is responsible for driving T cell specification by positively regulating the expression of the essential T cell factor, *Bcl11b*, at the transition of the T cell commitment stage [77].

The Notch-induced regulatory cascade triggers the activation of Bcl11b, which starts to be expressed at the T cell lineage commitment step occurring at the DN2a–DN2b transition [64,70]. The deficiency of this master regulator affects thymocyte maturation at the commitment checkpoint and impairs T cell receptor rearrangement [79,80]. Bcl11b induces a T cell-specific gene expression program that drives T-lineage commitment by limiting the cells from acquiring non-T cell fates. Specifically, Bcl11b blocks alternative lineages, such as natural killer, innate lymphoid cells and myeloid cells, as genes associated with stem/progenitor and non-T cell lineages are abnormally activated in absence of this master regulator [64,81,82,83]. Therefore, Bcl11b is an essential transcription factor that locks the cells in the T cell lineage by excluding access to other hematopoietic lineages and switching on T cell receptor rearrangement and the T cell program. Bcl11b is also highly expressed in innate lymphoid cells 2 (ILC2s) [84], which share the regulatory network driven by key transcription factors with T cells and thus, effector functionality [85,86]. Interestingly, despite the phenotypical and functional similarity among T cells and ILC2s, Bcl11b controls cell-type specific target genes by binding a distinct set of genomic regions and recruiting different protein complexes [87].

Pu.1 (encoded by *Spi1*) is a crucial player in the transcriptional regulation in uncommitted T cells, mostly myeloid cells. Its expression starts in the pre-thymic precursors, and it is silenced at the checkpoint of the T cell commitment, when Bcl11b is activated [50]. This hematopoietic transcription factor contributes to the establishment of cell lineage fidelity in myeloid, dendritic and B cells [88,89,90,91]. Nevertheless, Pu.1 is also required for the progression and survival of thymocytes in the early stages [92], and its expression pattern correlates with the transient capability of the cells to give rise to alternative lineages [93,94,95,96,97]. The forced expression of Pu.1 in committed T cells turns on myeloid genes and diverts the cells into macrophage and dendritic cells when Notch signaling is absent [66,98,99,100]. Endogenous Pu.1 mediates the positive and negative regulation of its target genes through a mechanism of re-deployment of other transcription factors such as Runx1 and Satb1 at stage-specific target loci [92,101]. Collectively, Pu.1 coordinates the core transcriptional network of uncommitted T cells by redirecting partner transcription factors.

The Runx transcription factor family regulates the transcriptional program of T cells at different developmental stages [102,103,104,105]. All the family members are initially expressed in the T cell progenitors; however, *Runx1* expression increases when *Runx2* and *Runx3* expression declines [67,106]. The activity of Runx1 has been mainly highlighted in the relationship with other key transcription factors such as Pu.1 and Bcl11b, and together they regulate the dynamic changes of transcriptional signatures before and after T cell commitment, respectively [83,101]. Nevertheless, Runx1 is not the only member of the Runx family to participate in the gene regulatory network of T cell development. Recent work dissected the importance of Runx1 and its paralog Runx3 in stage-specific deletion mouse models, identifying a functional redundancy in activating T cell genes and silencing alternative lineage genes when both factors are co-expressed [67]. Despite their continuous expression levels throughout T cell development, Runx1 and Runx3 control the transcriptome waves in a stage-dependent manner, and the dynamic redistribution of Runx proteins to stage-specific target loci is influenced by their interacting partners, Pu.1 before T cell commitment and Bcl11b in committed T cells [67,83,101]. Therefore, the Runx transcription factor family is required to shape the T cell gene regulatory network in a context-dependent manner.

## 5. Functions for Epigenetic Regulators in Early T Cell Development

### 5.1. Histone Modifiers

Transcription factors are not the only drivers of T cell commitment and differentiation, but the acquisition of distinctive, developmental-stage-specific transcriptomes is also dependent on correct epigenetic regulation (Figure 2; Table 1). Many epigenetic regulators are crucial for normal development, and their roles in T cell development have often been studied using conditional models driven by Cre-recombinase that acts at specific stages.

Polycomb repressive complex 1 (PRC1) and Polycomb repressive complex 2 (PRC2), which mediate the deposition of repressive histone marks, play important roles during T cell development [130], and mice deficient in Polycomb group genes exhibit T cell defects [131]. Among the PRC1 members, Bmi-1 has been shown to be involved in the regulation of immature T cells. Defective accumulation of thymocytes at the DN3 stage and cell death were observed in *Bmi-1* knockout mouse models, and it was shown that during the DN–DP transition, Bmi-1 was critical to regulate the suppression of p19Arf, which was found responsible for the cell cycle arrest [107]. The role of another PRC1 protein, Mel-18, was investigated in T cell development [108], and it was found that Mel-18 supported the expansion of early thymocytes and maintained the expression of *Hes1*, one of the Notch signaling target genes, in vitro and in vivo. The PRC1 members Ring1A and Ring1B mediate mono-ubiquitination of H2AK119 [132,133], and *Ring1A/B* double knockout resulted in a block at the DN3 stage and a strong reduction of TCRβ+ cells [113]. Interestingly, chromatin immunoprecipitation experiments identified Pax5, a master regulator of B cell development [134], as one of the main Ring1B targets, and deletion of *Pax5* was able to rescue T cell development, suggesting that PRC1 complex members were also involved in the suppression of the B cell lineage during thymocyte development [113].

H2AK119Ub can be removed by deubiquitinating enzymes, including BAP1 [135], MYSM1 [136] and ubiquitin-specific proteases (USP) such as USP16 and USP21 [30]. USP16 and USP21 are not involved in T cell development [137,138,139]. However, critical roles for Bap1 and Mysm1 in early thymocyte development have been reported. Conditional deletion of *Bap1* in T cells resulted in reduction of thymic cellularity and loss of DN thymocytes. Through the use of an in vitro differentiation system, it was further shown that Bap1 controlled cell cycle progression at the DN3 stage before the pre-TCR checkpoint through its histone de-ubiquitination function [114]. Severe T cell defects have also been observed in *Mysm1*-deficient mice [115,116], which was associated with Mysm1 function in the p53 pathway rather than its de-ubiquitinase activity [117].

EED, EZH2 and SUZ12 are components of the PRC2 complex [140], and several studies have evaluated the role of PRC2 proteins in late T cell differentiation and plasticity [141,142,143]. In addition, functions for these factors in early intrathymic lymphopoiesis have been reported. For instance, Eed contributes to the developmental progression at the β-selection checkpoint [109]. Ablation of *Ezh2*, the H3K27me3 writer, resulted in defective T cell development and abnormal distribution of the repressive mark in *Ezh*-deficient DN cells [110]. Moreover, the loss of Ezh2 and p53 deregulates the transcriptional program of T cell differentiation, leading to malignant transformation [144]. Interestingly, PRC2 interacts with Ikaros, a key factor that can act as repressor or activator in T cell development [145,146,147,148], and PRC2 binding to specific loci is dependent on Ikaros at the DN3 stage [149], suggesting a functional correlation between epigenetic regulators and transcription factors in early thymocytes.

The readers, writers and erasers of the active histone mark H3K4me are essential epigenetic enzymes with important functions in hematopoiesis [150]. In addition, they can contribute to the control of the T cell lineage. Cxxc1 is a key epigenetic regulator that specifically binds to unmethylated CpG-rich sequences and protects Polycomb-bound genes from DNA methylation in mouse embryonic stem cells [151]. In addition, Cxxc1 is a component of the COMPASS complex that contains the H3K4 methyltransferase Setd1 [152], and this association has been suggested to be critical for Cxxc1′s function in thymocyte development. Loss of Cxxc1 in a conditional mouse model was accompanied by a drastic reduction in thymic cellularity and an arrest at the DN3 stage [111], supporting a critical function of Cxxc1/Setd1 in T cell development.

The histone methyltransferase Setd2 catalyzes methylation at lysine 36 of histone 3 (H3K36me3) [153]. Conditional mouse models with loss-of-function mutations in *Setd2* resulted in reduced H3K36me3 and a partial arrest at the DN3 stage, during which Setd2 was required for the TCRβ rearrangement [154]. Consistent with this developmental block at the precursor stage, T cell-specific *Setd2* knockout mice showed an expansion of γδ T cells [112], suggesting a function of Setd2 in the early lymphocyte maturation.

Another histone modifier that was identified as a regulator of T lymphocytes was the protein arginine methyltransferase Carm1 [155]. Knockout murine models showed that this epigenetic factor promoted the differentiation at the transition from the DN1 to the DN2 stage [118,119], but the epigenetic changes driven by the arginine methyltransferase activity have not been investigated yet.

The reversible acetylation of lysine residues on histone tails is mediated by histone acetyltransferases (HATs) [156], including the CBP/p300 family of HATs [157]. The function of CBP/p300 has been elucidated in late T cell differentiation [158,159], but not much is known about their function during earlier stages. They acted as transcriptional coactivators, and deletion of either *CBP* or *p300* resulted in a reduction of DP thymocytes, but the double knockout showed a more drastic decrease in the number of mature T cells [120]. Mof is responsible for the global acetylation of H4K16 [160], and its deficiency leads to reduction of DP cells and consequently to a partial block at the DN3-DN4 transition, suggesting a primary role for this HAT in early T cells [121].

Histone deacetylases (HDACs) remove acetyl groups [161], and the roles for these erasers in developing T cells have been reported. While the deletion of *Hdac1* or *Hdac2* has no impact on thymocyte development, dual conditional deletion results in reduced thymic cellularity with a block at the transition to DP cells, causing a dramatic effect on thymocyte development [122]. This severe phenotype was accompanied by an increased acetylation of H3K9, demonstrating the requirements of Hdac1 and Hdac2 for chromatin regulation in the T cell identity [122]. Hdac3 acts at multiple stages during T cell development [162,163] and Cre-mediated *Hdac3* deletion causes an impaired progression of early thymocytes towards the DP stage, resulting in an abnormal accumulation of ISP cells that are not able to produce functional T cells [123].

### 5.2. DNA Methylation Machinery

Kramer and colleagues established an important role for Dnmt3a in the earliest T cell subsets by showing that its genetic ablation in murine hematopoietic stem cells provoked a significant accumulation of DN2 cells because of reduced apoptosis [124]. On the other hand, it was reported that a T cell-specific *Dnmt3b* knockout did not have an impact on T cell differentiation in a mouse model of MYC-induced lymphomagenesis [164]. Interestingly, knock-in mice with Dnmt3b mutations corresponding to the mutations found in immunodeficiency, centromeric instability, facial anomalies (ICF) syndrome patients [165] showed an increase of thymocyte apoptosis and an arrest at the DN to DP transition stage [125]. Reduced levels or the absence of serum immunoglobulins are characteristic for ICF syndrome. In addition, T cell defects have been described in ICF patients [166,167,168], but their contribution to the disease is incompletely understood. T cell proliferation and development was also dependent on Dnmt1 because impaired survival of TCRαβ+ cells and lower cell number of DP T cells were observed in *Dnmt1* conditional knockout mice [126].

The maintenance of DNA methylation requires UHRF1, which binds to hemi-methylated DNA and recruits DNMT1 [169]. The contribution of Uhrf1 to T cell lineage development was assessed in a conditional mouse model, suggesting a possible functional role in αβ and γδ T cell fate decisions due to its activity as epigenetic regulator [127].

T lymphopoiesis is impaired when the DNA methylation reader, Mbd2, is not expressed [128]. The deficiency in Mbd2 leads to an arrest at the DN3 stage and consequently, a reduction of the DP population. The T cell defect was linked to Mbd2′s function in regulating the expression of key genes of the WNT pathway, including *Tcf7*. As Wnt signaling has been shown to be crucial for proper T cell development [72,170], these studies have uncovered a link between an epigenetic regulator controlling early T cell development and the Wnt pathway. As reviewed extensively elsewhere [171,172,173], Wnt signaling is required for normal T cell development even though the targeted deletion of *beta* and *gamma-catenin* in thymocytes shows no phenotype because these mutations fail to eliminate canonical Wnt signaling. Other studies conclusively have shown the importance of Wnt signaling in early T cell development, as in the combined effects of the nuclear effectors of Wnt signaling, Lef1 and Tcf1 [174].

## 6. The Interplay of Epigenetic and Transcription Factors in Early T Cell Development

T cell development is built on decisions by progenitor cells that are instructed to differentiate into more mature T cells. The “instructions” that are controlling the development stages are not only external factors such as cytokines and environmental signals. Regulatory networks driven by stage-specific transcription factors constitute a key feature of the developmental changes during lymphopoiesis [10,175].

A specialized class of transcription factors, termed pioneer factors, can bind to their target sites within compacted chromatin [176], and this pioneering activity leads to increased DNA accessibility that allows the recruitment and binding of other transcription factors and chromatin remodelers [177]. The ability to trigger chromatin changes has been observed in lineage-determining transcription factors. For example, Pu.1 is a well-known pioneer transcription factor in myeloid cells [178,179]. A pioneering role of Pu.1 has recently also been described in uncommitted T cells, wherein Pu.1 is able to bind to its closed target sites and initiate the opening of chromatin [176,180]. Pu.1 can recruit two other transcription factors, Satb1 and Runx1, to genomic sites, and it can activate or repress genes by displacement of these transcription factors along the genome [101,181]. Downregulation of *Pu.1* expression is associated with the activation of Bcl11b, which defines the transition to committed cells and coincides with the loss of potential to develop toward myeloid lineages [82,92]. Activation of the *Bcl11b* gene requires the transcription of a non-coding RNA called Thymocyte Differentiation Factor (ThymoD) that facilitates enhancer–promoter communication [182]. Moreover, a computational model based on results from single-cell and bulk studies has been recently used to increase our understanding of the kinetics of T cell lineage commitment, elucidating the complex regulatory mechanism of Bcl11b activation that involves transcriptional and stochastic epigenetic control [183].

The transition from uncommitted to committed T cells also exhibits the largest transformation in chromatin accessibility and topologically associating domain (TAD) conformation [184], and this reorganization of the chromatin landscape correlates with changes in the distribution of genome-wide histone marks [11,185]. DNA sequences that become inaccessible after T cell commitment are highly enriched for a Pu.1 binding motif [11,69], and these sites lose the active histone mark (H3K4me2) and acquire the repressive modification (H3K27me3) [11]. Genomic loci that gain accessibility are bound by Bcl11b, and it seems that Bcl11b itself can contribute to maintaining long-range chromatin interactions of its target genes [184]. The two stage-specific transcription factors Pu.1 and Bcl11b are not the only factors that are able to modulate the chromatin landscape. Indeed, our data and others reveal that the master regulator, Tcf1, can coordinate chromatin accessibility in DN3 and DP T cells, unveiling a role of Tcf1 in the epigenetic landscape of T cells [68,69]. Moreover, Tcf1 exploits its intrinsic HDAC activity to regulate the gene expression in CD8 T cells [186], but it is still undiscovered whether the HDAC domain has a crucial function in the immature stages. Of note, the HDAC activity of Tcf/Lef factors is modest; it is much lower than conventional HDACs and needs high concentrations of inhibitors to be reduced. In addition, many splice variants of Tcf1 exist that do not contain the presumed HDAC activity but play roles in mature CD8 T cell differentiation [187], suggesting the involvement of additional mechanisms.

For instance, it is well established that master transcription factors and chromatin modifiers cooperate in directing B lymphocyte development [188,189]. Additionally, dysregulation of both epigenetic regulators and transcription factors induces immune lineage conversion [190]. While our knowledge about these processes in early T cell development is still limited, it is likely that epigenetic regulation is an essential component of T cell specification.

The progress toward mature T cells is orchestrated by the actions of chromatin modifiers [129], but the functional crosstalk among them and key transcription factors in early T cells remains poorly understood. Mouse models deficient for ATP-dependent remodeling enzymes suggested critical functions of these factors throughout T cell development [191]. In the context of early T cell development, Mbd3, a member of the NuRD complex, has an important impact on thymic lymphopoiesis [192]. Furthermore, it has been shown that a T cell specific knockout of Brg1/SMARCA4, which is the catalytic subunit of the SWI/SNF chromatin remodeling complex, displayed thymic defects associated with a developmental block at the DN stage [193]. Notably, *Cd4* and *Cd8* gene expression is coordinated by the combined functions of epigenetic regulators and transcription factors that ensure proper silencing in immature T cells [194]. The mechanism of recruitment and redistribution of epigenetic and transcription factors has been elucidated in a DN3-like cell line [83,181]. The master regulator of T cell commitment, Bcl11b, regulates the expression of its target genes by nucleation of chromatin remodeling complexes such as PRC1, NuRD, REST complexes, and Runx1 to specific genomic sites [83,87]. Bcl11b and its partners are able to modulate both activation and repression of genes, collaborating in this way toward the establishment of the T cell identity [83]. Overall, the identification of the complex interaction between chromatin modifiers and transcription factors is crucial for the understanding of the stage-specific transcriptional network and regulation of intrathymic cell fate determination. This is currently an area of intense investigation in several laboratories world-wide.

## 7. Concluding Remarks

The development of T lymphocytes from uncommitted progenitor cells in the thymus is a highly regulated process. Regulation involves external signals such as cytokines, Wnt, BMP and Notch signals as well as cell-intrinsic mechanisms including epigenetic control. It is therefore not surprising that deletion of fairly ubiquitous epigenetic mediators (HDACs, SWI/SNF factors, etc.) in specific stages of T cell development leads to profound functional defects. In cases where effects are not found, there is likely a molecular redundancy. It should also be noted that in many studies on epigenetic factors, conditional knockout systems are used, most often employing Lck-Cre, which is not active until the DN3 stage. Hence, functional effects in earlier stages of T cell development may not have been discovered yet. More interesting, therefore, are lineage-specific regulators or epigenetic factors that show strong differential expression over the various stages of development, as differential expression may imply an important function. A key issue is the physical and functional interaction between pioneering transcription factors and epigenetic regulators, and especially epigenetic readers. For instance, some major transcription factors may mediate changes in the epigenetic state because they are apparently capable of setting up a specific lineage as was shown for Tcf1, possibly by intrinsic HDAC activity [186]. On the other hand, other examples indicate that epigenetic regulation is superimposed on the actions of lineage-specific transcription factors. A fascinating example is hematopoiesis and T cell development in *Wnt3a*-deficient mice. In *Wnt3a*^−/−^ mice, there are fewer hematopoietic stem cells that remain capable of multilineage differentiation but lack self-renewal, as shown in secondary transplantations [170]. Interestingly, transplantation of *Wnt3a*^−/−^ hematopoietic stem cells (HSCs) in *Wnt3a*-proficient hosts does not rescue this phenotype. Apparently, there is a window of time in development in the fetal liver during which Wnt3a needs to be present; otherwise, HSCs do not form properly. This is likely due to an epigenetic regulation of the stem cell fate in this time window. Indications for a link to aberrant epigenetic regulation come from RNA-seq experiments on *Wnt3a*-deficient HSCs, showing sharply reduced expression of genes involved in epigenetic regulation (Staal, unpublished). Thus, this represents an example of an extrinsic effect on epigenetic regulation due to the temporal requirement for Wnt3a signaling by a neighboring cell. How pioneering transcription factors and epigenetic regulators interact and set up lineage specific gene expression programs is currently largely unknown. Given that various developmental checkpoints (T lineage commitment, β selection, positive and negative selection, CD4 vs CD8 division) are well defined functionally and phenotypically, and individual cells can be readily purified for further study, T cell development forms an ideal model to investigate these fascinating questions.

## Figures and Tables

**Table 1:** Phenotypes of epigenetic factor-depleted murine models.

	Epigenetic Regulator	Mouse Model	Phenotype	Reference
Histone modifiers	Bmi-1 (PRC1)	KO	Block at DN3b stage, reduced thymic cellularity	[107]
	Mel-18 (PRC1)	KO	Increased cell death at DN1/DN2 stage, reduced thymic cellularity	[108]
	Ring1A/B (PRC1)	*Ring1A* KO/CD4-cre, *Ring1B* KO	Block at DN3a stage, failure to repress B cell lineage	[109]
	Bap1	Cre-ERT2	Block at DN3a stage, reduced thymic cellularity	[110]
	Mysm1	KO	Block at DN1 stage, reduced thymic cellularity	[111,112,113]
	Eed (PRC2)	hypomorphic *Eed* allele	Block at DN3a stage, reduced thymic cellularity	[114]
	Ezh2 (PRC2)	Mx1-cre	Block at DN2 stage, no proliferative impairment	[115]
	Cxxc1	hCD2-cre, Lck-cre, Cre-ERT2	Block at the DN–DP transition, reduced thymic cellularity	[116]
	Setd2	Mx1-cre, Lck-cre	Block at DN3a stage, reduced thymic cellularity	[117]
	Carm1	KO	Block at DN1 stage, reduced thymic cellularity	[118,119]
	CBP/p300	Lck-cre	Strong reduction of DP cells	[120]
	Mof	Lck-cre	Block at DN3–DN4 transition stage, reduced thymic cellularity	[121]
	Hdac1/Hdac2	Lck-cre	Block at DN–DP transition, reduced thymic cellularity	[122]
	Hdac3	Lck-cre	Block at ISP stage	[123]
DNA methylation machinery	Dnmt3a	Mx1-cre	Block at DN2 stage	[124]
	Dnmt3b	ICF1 mutation knock in	Block at DN–DP transition, reduced thymic cellularity	[125]
	Dnmt1	Lck-cre	Block at DN3b stage, reduced thymic cellularity	[126]
	Uhrf1	Lck-cre	Block at ISP stage, reduced thymic cellularity	[127]
	Mbd2	KO	Block at DN3 stage, reduced thymic cellularity	[128]

Abbreviations: KO: knockout; DN: double negative; ISP: immature single positive; DP: double positive. Inspired by review [129].
